# *In-situ* bio-stimulation for enhanced biological methane production and its effect on the microbiome of CBM wells in Raniganj block, India

**DOI:** 10.3389/fbioe.2025.1571653

**Published:** 2025-06-27

**Authors:** Mansi Chawla, Meeta Lavania, Nishi Sahu, Dipanjana Banerjee, Nimmi Singh, Banwari Lal

**Affiliations:** Environmental and Industrial Biotechnology Division, The Energy and Resources Institute, New Delhi, India

**Keywords:** coalbed methane, *in situ* biostimulation, nutrient amendment, biogenic methane enhancement, microbiome analysis

## Abstract

Microbially enhanced coalbed methane production (MeCBM) is a way towards translation of the recent momentum of the high demand for renewable energy into operational capacity. The present study demonstrates the enhancement of biogenic methane in coalbed methane (CBM) wells of an Indian coal reservoir via *in-situ* biostimulation. A laboratory-scale strategy was previously developed to understand and enhance the microbial processes for the bioconversion of coal to methane before transferring it to the field. The quantitative measurement of gas production after the industrial-scale microbial stimulation job carried out at the EOGEPL Raniganj block indicated upto a four-fold enhancement in methane production, with the best results observed in Well-B, from a baseline production of 117.04 standard cubic meters per day (scmd) to 461.38 scmd, followed by Well-E, with an increase from 210.93 scmd to 385.19 scmd, and Well-C, with an increase from 514.22 scmd to 670.22 scmd. Molecular and isotopic compositions of the gases collected by post-nutrient injection have been studied and the results indicate the occurrence of secondary microbial gas. The 16s rRNA amplicon sequencing analysis of formation water samples post-nutrient injection, and its comparison with previously published pre-injection microbial community analysis gives an insight into the impact of the microbial stimulation on the indigenous microbiome of the CBM wells. The present study provides a framework for understanding the effects of *in-situ* biostimulation via nutrient amendment in a coal reservoir. Further, the findings of the study will help to implement methane enhancement strategy via biostimulation on a wider range of coal fields to enhance its commercial potential.

## 1 Introduction

Coalbed methane (CBM) is an unconventional natural gas that has gained worldwide interest. Its calorific value and CO_2_ emissions are comparable to those of conventional natural gases (R. Chen et al., 2023). The environmental consequences of coal-fired power plants can be greatly mitigated by increasing the use of this unconventional gas source (Dresselhaus and Thomas, 2001; McJeon et al., 2014), which has led industrial corporations to concentrate on improving the production of biogenic coalbed methane.

While thermogenic methane is created during the coalification process, biogenic methane is the result of continuous microbial coal degradation. The biogenic coal-to-methane conversion is a multi-step process that involves microbial communities including a diversity of both bacterial and archaeal groups. Complex carbon in coal is progressively broken down by hydrolytic bacteria into intermediate organic compounds such as polyaromatic hydrocarbons, monoaromatic carboxylic acids, single-ring aromatics, long-chain alkanes, ketones, long-chained fatty acids, etc. . These organic are then broken down by fermentative bacteria into substrates such as CO_2_, H_2_, volatile fatty acids, methanol, methyl sulfide, *etc.*, which the methanogenic archaea use for methane gas production (Colosimo et al., 2016; Jones et al., 2010; Orem et al., 2010). The volatile fatty acids produced during the fermentation stage can be utilized by acetogenic bacteria, forming CO_2_ and H_2_ (Beckmann et al., 2011) that hydrogenotrophic methanogens use during the methanogenesis step (Equation 1). On the other hand, homoacetogenic bacteria can convert CO_2_+H_2_ to acetate which can be utilized by acetoclastic methanogens to produce methane (Equation 2), while methylotrophic methanogens directly use methanol and other methylated compounds as substrates for methane production (Equation 3) (Guo et al., 2012a; Guo et al., 2012b; Laguillaumie et al., 2023; Thauer, 1998; Buan, 2018).

$${\text{CO}}_{2}+4H_{2}\;\to \;{\text{CH}}_{4}+2H_{2}O∆G=-131\text{ kJ}/\text{mol},\text{hydrogenotrophic reaction}$$ (1)

$${\text{CH}}_{3}\text{COOH }\to \;{\text{CH}}_{4}+{\text{CO}}_{2}∆G=-31\text{ kJ}/\text{mol},\text{acetoclastic reaction}$$ (2)

$$4{\text{CH}}_{3}\text{OH }\to \;3{\text{CH}}_{4}+{\text{HCO}}_{3}^{-}+H_{2}O+H^{+}∆G=-105\text{ kJ}/\text{mol},\text{methylotrophic reaction}$$ (3)

Research on the microbial composition of various CBM reservoirs has revealed the presence of diverse communities of both bacteria and archaea (Green et al., 2008; Midgley et al., 2010; Singh et al., 2012; Strąpoć et al., 2008), with the bacterial diversity being higher than the archaeal diversity (Barnhart et al., 2013; He et al., 2020; Y. Li et al., 2023; Penner et al., 2010). This suggests that the *in-situ* microbial communities work in syntrophic action, with coal hydrolyzing and fermentative bacteria breaking down complex coal, rendering substrates available for the methanogenic populations for the final step of methanogenesis (Iram et al., 2017; Strąpoć et al., 2011). The bacterial communities have been seen to be dominated by coal hydrolyzing Proteobacteria and Actinobacteria, with Firmicutes, known for acidogenic capabilities, often as a minority. The dominance of Proteobacteria in most CBM wells is noteworthy due to its known association with methanogens, and the capability to degrade Polycyclic Aromatic Hydrocarbons (PAH) (Guo et al., 2012a; Meslé et al., 2013; Penner et al., 2010). Despite being a minority in the *in-situ* microbial composition of some of the studied CBM reservoirs. The members under phylum Firmicute, particularly fermentative and acetogenic bacteria, are an important part of the methanogenic community, and may be enriched in the laboratory scale microcosms by supplementing nutrients for microbially enhanced methane production (Li et al., 2008; Ritter et al., 2015).

With the discovery of the biogenic CBM synthesis processes, attempts have been made to boost CBM production by using techniques such as biostimulation and bioaugmentation. *In-situ* biostimulation is performed by adding organic nutrients, trace elements, inorganic minerals, yeast extract, etc. To stimulate or activate the microbial population in a CBM field (R. Chen et al., 2023). Nutrient amendment in the coal field supports the growth of the microbial community and encourages metabolic processes, which include the breakdown of coal into methane (Park and Liang, 2016). Table 1 shows cases of enhancement in methane yield achieved in previous studies via nutrient addition.

**TABLE 1:** Previous studies showcasing methane yield enhancement via nutrient addition.

Study	Treatment	Maximum CH_4_ Yield
Bi et al. (2017)	Formation water-based nutrient recipe	1,042 ft^3^/ton
Harris et al. (2008)	H_2_/CO_2_ amendment Inorganic nutrient amendment	4.5–12.0 scf/ton 4.2–9.1 scf/ton
Davis and Gerlach (2018)	Organic amendment (Algae, Cyanobacteria, yeast cells and granulated yeast extract)	2,185 μg CH_4_/g coal
J. Zhang et al. (2015)	Variable strengths of MS medium	111 ft^3^/ton
Barnhart et al. (2017)	Modified anaerobic co-culture medium (CCM) with coal	311 ± 51 μg CH_4_/g coal
	Modified anaerobic co-culture medium (CCM) with coal + Yeast Extract	1,052 μg CH_4_/g coal
	Modified anaerobic co-culture medium (CCM) with coal + Algal Extract	576 μg CH_4_/g coal
B. Liu et al. (2024)	Bituminous coal + Modified enrichment media	243.3 μmol/g coal
	Anthracite coal + Modified enrichment media	207.3 μmol/g coal
	Coking coal + Modified enrichment media	163.1 μmol/g coal
Zhou et al. (2022)	Anaerobic culture medium	5.75 m^3^/t
Rathi et al. (2015a)	Modified MSP medium	22.9 mM/g coal

Bioaugmentation is performed by introducing specific microbes involved in the process of methane generation into the system along with nutrients for enhancement of methane production. Previous works have demonstrated significant enhancement in methane yield by using mixed methanogenic cultures including bacteria capable of hydrocarbon degradation, and methanogens responsible for direct conversion of substrates to methane. Jones et al. (2010) studied the effect of bioaugmentation treatment on methane yield by adding a microbial consortium that included bacteria such as *Acinetobacter* sp.*, Azonexus sp*., and *Pelotomaculum,* along with methanogens, particularly, *Methanosaeta concilii* and *Methanosarcina* spp., and *Methanomicrobiales*, which showed a significant enhancement in methane production in the microcosm. Gállego-Bravo et al. (2023) studied the production of methane from organic waste via anaerobic digestion and demonstrated an improvement in methane yield by 4% by bioaugmenting the anaerobic digestor with hydrogenotrophic methanogens, dominantly Methanoculleus.

The productivity as well as longevity of a CBM well can be improved by such microbial interventions, which could improve the economics and viability of produced gas from CBM wells, thereby making it a sustainable process. A few industrial corporations have attempted to increase the production of biogenic CBM on a commercial level using biostimulation techniques. Luca Technologies, Inc. Implemented their methodology of nutrient addition at four U.S sites: Black Warrior Basin, Uinta Basin, San Juan Basin, and Powder River Basin (Strąpoć et al., 2011). The nutrient mixture contained weak organic acids, glycerol, complex nutrients like yeast extract, and synthetic vitamins and minerals (Ulrich and Bower, 2008). Studies have confirmed that glycerol acts as an easily digestible co-substrate that increases methane production (Hutňan et al., 2013; X. Li and Shimizu, 2023). Organic acids, and vitamins and minerals act as substrates biomethane production (Ritter et al., 2015). Weak organic acids can be directly utilized by methanogenic archaea that produce biomethane (Liu et al., 2022; Wang et al., 2019). Ciris Energy also implemented *in-situ* biostimulation in the Powder River Basin using synthetic nutrients and yeast extract (Ritter et al., 2015). Addition of yeast extract improves the carbon/nitrogen ratio and enhances biomethane production due to the presence of yeast nucleotides, one of the major components in yeast extract, which can help microbes digest and absorb nutrients at a higher rate, thus enhancing their growth performance (Zhang and Liang, 2017). Nutrient supplementation with yeast extract in coal experiments has previously demonstrated significant enhancement of methane in the laboratory. Zhang and Liang (2017) observed that reducing the concentration of yeast extract from 2 g/L to 0.5 g/L in nutrient media having coal had a negative impact on the production of methane, with the production levels decreasing from 200 ft^3^/ton to 50 ft^3^/ton. Similarly, the study performed by (Davis et al., 2018) demonstrated that in contrast to the 0.5 g/L yeast extract amended coal treatments, which ranged from 1960 to 2,185 μg CH_4_/g coal, the methane concentrations for the 0.1 g/L yeast extract amended coal treatments ranged from 1,371 to 1,456 μg CH4/g coal. (Barnhart et al., 2017). production of methane in sets with media + coal and media + coal + yeast extract at day 165 as 341 ± 89 μg and 1,400 ± 313 μg respectively.

The present work is a field-scale implementation of the *in-situ* biostimulation technique for enhancement of coalbed methane production was performed in CBM wells located in the Raniganj coal-seam reservoir of the Gondwana Basin, India. The objective of the study was to assess the impact of *in-situ* biostimulation via nutrient injection on the indigenous microbial communities of the formation water. The bottom-hole temperature of the selected CBM wells ranges between 55°C and 65°C, with abundance of thermophilic microbial communities in the reservoir. The nutrient treatment strategy was developed according to the biogeochemical and microbiological assessment in the feasibility study performed in a prior investigation (Chawla et al., 2023).

## 2 Materials and methods

### 2.1 Geology of the study site and characteristics of the study well

India offers great potential for CBM exploration and development because it contains the world’s fifth-largest coal reserves. The majority of India’s coal reserves and all of the country’s present CBM producing blocks are found in the Gondwana sediments in Eastern India. A significant portion of the most promising regions for CBM development and production are in the Son and Damodar valleys in Eastern India. The Raniganj block is located in the Burdawan district of West Bengal, India, spread over 500 sq. km in the Damodar valley basin with thick Permo-Carboniferous Gondwana coal seams. The Barakar and Raniganj Formations have commercial coal resources in the Raniganj Coalfields. The Barakar Formations are made up of riverine sediments that were deposited with a regionally variable thickness of coal seams that are associated. However, only a very small portion of this coalfield exposes the coal seams of the Barakar Formation. With a thickness of almost 1,000 m, the Raniganj Formation is most developed there. In the Raniganj Formation, ten regional coal seams with an average thickness of more than 1.2 m are identified. In the Raniganj Formation, the lower seams are comparatively thicker. The eastern portion of the coalfield contains the laterite and alluvium outcrops of the Raniganj measures (Chattaraj et al., 2021).

**FIGURE 1:**
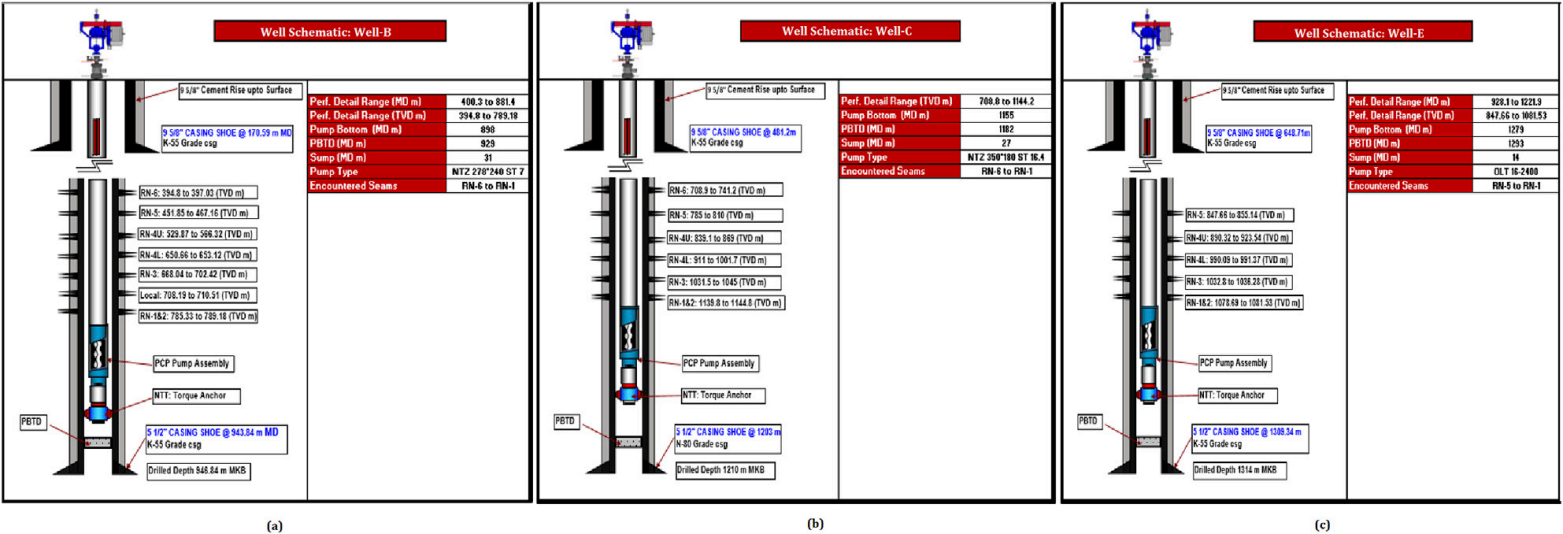
Schematic representation of the study wells: **(a)** Well-B, **(b)** Well-C, **(c)** Well-E.

CBM Well-B having a drilled depth of 946.84 m, profile depth of 881.4 MD m, net pay of complete coal seam 24.86 TVD m, and coal seam interval 30.30 MD m (Figure 1a), CBM Well-C having a drilled depth of 1,210 m, profile depth of 1,144.2 MD m, net pay of complete coal seam 28.3 TVD m, and coal seam interval 26.3 MD m (Figure 1b), and CBM Well-E having a drilled depth of 1,314 m, profile depth of 1,221.9 MD m, net pay of complete coal seam 21.73 TVD m, and coal seam interval 27.4 MD m (Figure 1c) were selected from the Eastern block of the Raniganj coal field. The wells are lined with a K55 grade casing shoe secured by concrete. The average bottom-perforation temperature is 49°C. Table 2 provides detailed parameter descriptions of the wells.

**TABLE 2:** Detailed parameters of the Study Wells.

S.No	Parameters description	Results		
		Well-B	Well-C	Well-E
1	Bottom Perf (MD m)	881.4	1,144.2	1,221.9
2	Bottom Perf (TVD m)	789.81	1,144.2	1,081.54
3	PBTD (MD m)	929	1,182	1,293
4	Sump (MD m) From Bottom Perf	48	38	71
5	Temp Deg C @ Bottom Perf (TVD m)	49	59	57
6	Hydro Static Pressure (Psia)	1,122	1,625	1,536
7	Injectivity (LPM)	7.6 (Pre-frac)	NA	NA
8	Permeability (Pre-Frac) (md)	4.8 (IFT)	NA	NA
9	Fracture Half Length (m)	57	114	29
10	Permeability (Post Frac) (md)	4 (HM)	3 (HM)	7 (HM)
11	Volume of sump (L)	143	466	214
12	Volume of the Casing (excl. Sump) (L)	10,868	14,108	15,066
13	Volume of Tubing (L)	2,653	3,444	3,678
14	Tubing Displacement (L)	1,031	1,338	1,429
15	Volume of Annulus (Casing-Tubing) (L)	7184	9326	9959
16	Net Pay of Completed Coal Seams (TVD m)	24.86	28.30	21.73

*NA-Not Available.

### 2.2 Feasibility studies

Several factors should be taken into account before implementing Microbially Enhanced Coalbed Methane (MeCBM) tactics that have been studied in laboratories in an actual setting. A deeper knowledge of every stage of the coal-to-methane conversion process is made possible by bench-scale experiments, which are significantly simplified models of the subterranean coal environment and offer more control over the conditions. Understanding the *in-situ* reservoir conditions in regions where active microbial CBM generation occurs is necessary to comprehend the potential effects of varying natural conditions on methane generation rates associated with MeCBM projects.

The formation water and coal samples collected from the CBM wells were previously analyzed for physicochemical composition as part of the feasibility study (Chawla et al., 2023). Enrichment of the methane producing microbial consortium in the formation water was also performed in methanogen-specific modified MPB media. For the *in-situ* biostimulation of the indigenous microbial diversity involved in the biological enhancement of CBM production, Well-B, Well-C, and Well-E were selected for field trial in accordance with the results of feasibility study and availability of wells for field job (Chawla et al., 2023). To ascertain whether the indigenous microbial population of the studied CBM wells would utilize coal as a carbon source for biological methane production was also tested on a lab-scale in the initial phase of the study during media modification experiments in the feasibility studies (Supplementary Figure S1). A single factor experiment was performed during the media optimization studies to identify the essential media components for microbial methane production Supplementary Figure S2). Production of Volatile Fatty Acids (VFA) was also analyzed during the enrichment process (Supplementary Table S1), with results indicating anaerobic digestion of coal and subsequent utilization of VFA during methane formation.

#### 2.2.1 Compatibility study

Three treated technical water samples, namely, TW-1, RO-64, and RO-50 were collected from water sources near the well site and tested for compatibility with the modified nutrient formulation of MPB media: K_2_HPO_4_; 0.4 g/L, KH_2_PO_4_; 0.2 g/L, MgCl_2_.6H_2_O; 0.1 g/L, NaCl; 0.5 g/L, Yeast Extract; 0.5 g/L, NaHCO_3_; 0.2 g/L, NH_4_Cl; 0.2 g/L (Chawla et al., 2023). Three sets of modified MPB media were prepared with technical water samples to check for biogenic methane production. The media sets were prepared in anaerobic serum bottles. 1% (w/v) coal was added as a carbon source, and the bottles were sparged with nitrogen to maintain an anaerobic environment, followed by inoculation of each technical water set with 1% (v/v) formation water collected from each well. The pH of all the sets of media was maintained at 7.5. The bottles were incubated at 55°C for 30 days. Control sets consisted of media prepared with technical water samples, and added coal (1% (w/v)). To stimulate the coal well conditions, no agitation was used during the experiment at the lab-scale. The C/N ratio of the coal received from Well B, Well C, Well E, and yeast extract are 41.55, 51.54, 52.56, and 3.34 respectively.

Methane production was monitored after 30 days of incubation by extracting 0.5 mL of headspace gas samples from the anaerobic serum bottles using a gas-tight syringe and quantifying the produced methane using gas chromatography. The gas analysis was carried out for triplicate bottles of each technical water set. The data points represent the triple average plus standard deviation (<5% of average).

### 2.3 Demonstration of microbially enhanced CBM in the Raniganj coal field

#### 2.3.1 Establishing a production baseline prior to nutrient injection

Daily gas and water production trends of the wells were monitored for 1 month before the field demonstration. Average gas production rates of Well-B, Well-C and Well-E were calculated, and the baseline values of gas production were set as 117.04 scmd, 514.225 scmd, and 210.193 scmd respectively, with baseline water production of 5.26 cmd, 10.8 cmd, and 16.67 cmd respectively.

#### 2.3.2 Nutrient injection set-up

An optimized nutrient media of composition: K_2_HPO_4_; 0.4 g/L, KH_2_PO_4_; 0.2 g/L, MgCl_2_.6H_2_O; 0.1 g/L, NaCl; 0.5 g/L, NH_4_Cl; 0.2 g/L, NaHCO_3_; 0.2 g/L, and Yeast extract; 0.5 g/L, previously formulated according to the abundance of thermophilic microbial communities in the selected CBM wells, and tested by Chawla et al. (2023) during the feasibility studies was used for nutrient injection in the CBM wells. Treated technical water selected according to the results of the compatibility studies was collected from the well site shortly before the injection and used for nutrient preparation. The total injection volume was 200 m^3^ for each well, prepared in thirty non-corrosive and sterilized water tanks (20 m^3^ each to avoid contamination). An injectivity test was performed in Well-B, Well-C and Well-E to establish an injectivity rate of 2.11 barrels per minute (BPM), 2.4 BPM, and 2.15 BPM respectively. A pumping unit was placed to inject the nutrient solution into the well as per the injectivity rate. 197 m^3^ nutrient solution was injected in each well, followed by 3 m^3^ water filled in the columns after injection. The Casing Head Pressure (CHP) in the well-head was at 0 PSI throughout the injection process, after which the wells were closed and kept on incubation for the period of 30 days.

### 2.4 Post-job monitoring

After an incubation period of 30 days, the wells were opened and dewatered to gradually knock out the amount of nutrients injected into the wells, i.e., 200 m^3^ in each well during the field demonstration. After a complete water knockout, the wells were ready to be closely monitored for post-injection assessment. Formation water and gas samples were then collected for analysis. Gas was collected from the well-heads in nitrogen-flushed cylindrical metal gas bombs. The formation water samples were collected in pre-sterilized anaerobic serum bottles containing 2% Na_2_S. The bottles were filled up to the brim and sealed while avoiding any air bubbles (Rathi et al., 2015b). The samples were transported to the laboratory in under 48 h, stored at 4°C, and were immediately processed for all required analyses.

#### 2.4.1 Physicochemical characterization of formation water

The formation water samples collected from the CBM wells after well opening were analyzed for physicochemical characterization. The analysis was performed according to the American Petroleum Institute (API) standard and APHA guidelines. The samples were analyzed for total iron content, sulphate, fluoride, calcium, magnesium, sodium, potassium, and nitrate. The parameters of analysis included heavy metal concentrations of arsenic, chromium, lead, cadmium, zinc, nickel, manganese, copper, silver, and mercury, along with the pH, electrical conductivity, and total dissolved solids. Formation water samples were collected bi-monthly in three sampling phases to study physicochemical characterization.

#### 2.4.2 Gas and water production trends

The gas and water production trends were measured via an online gas and water flowmeter installed on the well pads, which collected per-day and cumulative data for post-injection monitoring. A digital platform interface, Sensia Avalon, was deployed for the analysis and digitalization of well-related activities.

#### 2.4.3 Stable carbon isotope analysis

Stable carbon isotope study is an important analytical tool that has been used in this study to identify the genesis of the CBM gas. Comprehending the source and nature of CBM gas being produced in the wells after the microbial stimulation job is a critical parameter in this study, as it corroborates the results of microbial stimulation in the reservoir. As per the methodology described by Wang et al. (2019), the Keshava Deva Malaviya Institute of Petroleum Exploration, Oil and Natural Gas Corporation Ltd. analyzed the molecular and isotopic compositions of the gas samples collected on opening the wells after the biostimulation field trial.

#### 2.4.4 Genomic and data analysis of microbial diversity

The formation water samples collected from the wells in the third sampling round after the microbial stimulation job were processed for extraction of genomic DNA. The samples were filtered using a filter membrane having a pore size of 0.22 μm, and particles suspended in the formation water remained on the filter membrane. The DNeasy PowerWater Kit (Qiagen, Germany) was used for DNA extraction following the protocol provided by the manufacturer. The quantitative and qualitative analysis of the extracted DNA was performed using Thermo Scientific NanoDrop 2000 spectrophotometer. Sequencing was carried out on the high-quality extracted DNA samples that showed an A260/280 ratio between 1.8 and 2.0 and concentrations higher than 50 ng/μL.

The extracted DNA samples were processed for amplicon sequencing by amplifying the V3-V4 region of the bacterial 16S rRNA gene using primers 341F (5′-CCTACGGGNGGCWGCAG-3′) and 785R (5′-GACTACHVGGGTATCTAATCC-3′). In order to understand the overall diversity of the microbial population. V3-V4 amplicon sequencing was carried out on the Illumina MiSeq 2,500 platform by Medgenome Pvt. Ltd.FASTQC tool v 0.11.8 (Babraham bioinformatics) verified the quality of sequences after demultiplexing and adaptor/primer/barcodes sequence removal from raw reads. FLASH v 1.2.11 software was used to merge the paired-end readings of every sample (Magoč and Salzberg, 2011). The QIIME 1 standard protocol was adhered to, as stated in Quantitative Insights Into Microbial Ecology (López-García et al., 2018). Operational Taxonomic Units (OTU) picking was done against SILVA database version 132. The microbial analysis was done in the software MicrobiomeAnalyst (Dhariwal et al., 2017). The metagenome sequence reads of the samples have been submitted to the NCBI archive under Bioproject accession number PRJNA1119685.

The Alpha diversity analysis using the Chao1 diversity index, and the prediction of functional profiles of bacterial taxa based on the 16s rRNA sequencing were performed on Metagenassist software using the amplicon sequencing data according to the Silva-132 database. The rarefaction curve plotted between species richness and sequence sample size using MicrobiomeAnalyst has been shown in Supplementary Figure 3.

## 3 Results and discussion

### 3.1 Compatibility of the well-site technical water with the designed nutrient formulation

Compatibility studies were performed with technical water samples RO-50. RO-064 and TW-1, to check the suitability of these water sources for the application of biostimulation technology at field scale. The water samples were sourced from a reverse osmosis water system, having pH between 6.5 and 7, maximum specific conductivity of 10 μS/cm at 25°C, 1–2 mg/L total solids, and a maximum silica content of 1 mg/L (according to ISO 3025). No precipitation or turbidity was observed during nutrient preparation, indicating that all technical water samples received from the well-site are compatible with the modified nutrient media designed for the field trial. Moreover, the quantification via gas chromatography of methane gas produced in the headspace of the incubated serum bottles confirms the potential of the nutrient media prepared with technical water samples for supporting the growth of a specific methanogenic consortium that can use the bituminous coal found in thermogenic CBM reservoirs as a carbon source to facilitate *in-situ* methane enhancement.

**FIGURE 2:**
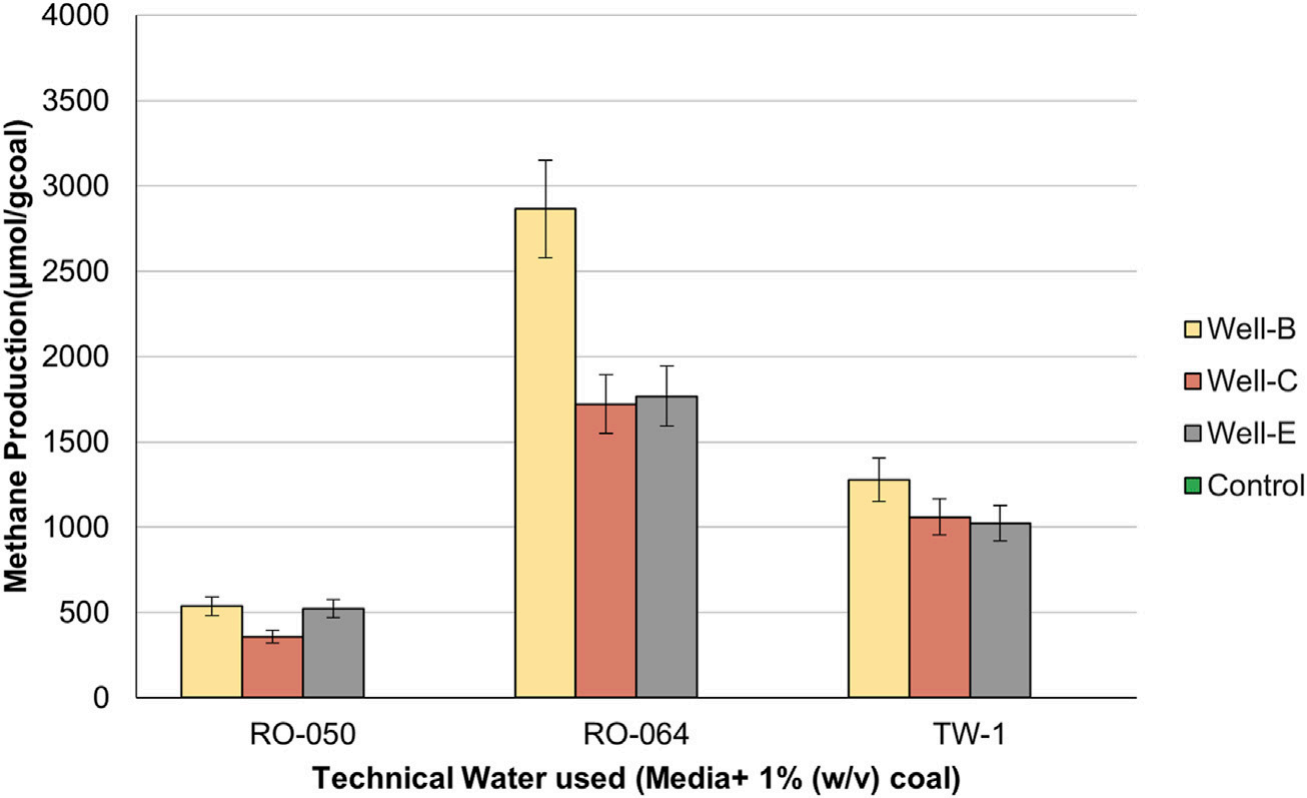
Methane production in the headspace of the serum bottles containing nutrient media prepared with technical water samples.

Figure 2 represents the amount of methane gas produced in the headspace of serum bottles containing nutrient media prepared with RO-50, RO-064, and TW-1. Each technical water sample was tested using formation water samples from Well-B, Well-C and Well-E as inoculums. In the case of RO-064, 2,864.7 μmol/g coal, 1720.22 μmol/g coal, and 1768.36 μmol/g coal of methane was produced in the headspace of media bottles inoculated with formation water collected from Well-B, Well-C, and Well-E respectively, which was higher than other technical water sets. The control sets showed no methane production.

Therefore, it could be concluded that technical water RO-064 showed the most compatibility and potential to support the targeted microbial consortium for enhanced methane production. In accordance with the results of the compatibility study, technical water RO-064 was selected for the preparation of the nutrient media to be injected into the well.

### 3.2 Post-injection monitoring

#### 3.2.1 Physicochemical characterization

Physicochemical analysis was performed on the formation water samples was first collected from the treated wells after complete nutrient media knockout, followed by second and third phase samples collected on a bi-monthly basis (Table 3). The results show that the amount of some heavy metals including cadmium, zinc, chromium, and silver consistently below the detectable limit (<0.01). The concentrations of all parameters fall in a close range when compared amongst three sampling phases, indicating a stable environment for the microbes throughout the monitoring period. The pH of Well-B formation water samples collected in sampling phases 1, 2 and 3 were 8.4, 8.8 and 8.6, respectively, and the total dissolved solids were observed as 2,557 mg/L, 2,658 mg/L and 2,634 mg/L, respectively. The electrical conductivity of the samples from sampling phases 1, 2 and 3 was found to be 5.51 m/cm, 5.39 m/cm and 5.38 m/c, respectively. The pH of Well-C formation water samples collected in sampling phases 1, 2 and 3 were 8.2, 8.3 and 8.2 respectively, and the total dissolved solids were observed as 3,726 mg/L, 4089 mg/L and 3,992 mg/L respectively. The electrical conductivity of the samples from sampling phases 1, 2 and 3 was found to be 15.21 m/cm, 12.65 m/cm,and 12.91 m/cm respectively. The pH of Well-E formation water samples collected in sampling phases 1, 2 and 3 were 8.5, 8.3 and 8.3 respectively, and the total dissolved solids were observed as 2,743 mg/L, 2,568 mg/L and 2,677 mg/L respectively. The electrical conductivity of the samples from sampling phases 1, 2 and 3 was found to be 5237 μS/cm, 5498 μS/cm,and 5373 μS/cm respectively. The medium-basic pH range has been considered optimal for methanogenesis (K. Wang et al., 2014). pH is a key factor that affects the production of VFA during the fermentation step. Y. Chen et al. (2013) determined that pH around 8.0 is optimal for VFA production during co-fermentation of food waste. Jin et al. (2019) studied the effect of pH anaerobic fermentation by monitoring the VFA composition and concentrations under variable pH and analyzing the changes in the microbial community via 16s rRNA sequencing. Slightly alkaline pH and thermophilic conditions may lead to higher accumulation of acetic acid, a crucial substrate in the methanogenesis step.

**TABLE 3:** Physicochemical analysis of the formation water sample.

S.no	Parameter	Test method	Results (mg/L)								
			Well-B			Well-C			Well-E		
			1st sampling phase	2nd sampling phase	3rd sampling phase	1st sampling phase	2nd sampling phase	3rd sampling phase	1st sampling phase	2nd sampling phase	3rd sampling phase
1	Cadmium (Cd)	APHA-3100 (B)	<0.01	<0.01	<0.01	0.04	0.01	0.01	0.01	<0.01	<0.01
2	Arsenic (As)	IS:3,025 (P-37):1988	0.043	0.020	<0.01	0.083	0.046	<0.01	0.033	<0.01	<0.01
3	Zinc (Zn)	APHA-3100 (B)	<0.01	<0.01	<0.01	<0.01	0.023	<0.01	0.057	0.06	0.059
4	Total Chromium (Cr)	APHA-3500 (B)	<0.01	<0.01	<0.01	<0.01	<0.01	<0.01	<0.01	<0.01	<0.01
5	Nickel (Ni)	APHA-3111(B)	0.19	<0.01	0.05	0.06	0.048	0.12	0.037	0.06	0.055
6	Total Iron (Fe)	APHA-3100(B)	4.50	5.62	0.53	4.41	7.62	5.7	9.12	7.45	7.61
7	Copper (Cu)	APHA-3111 (B)	0.05	0.12	<0.01	0.04	0.03	0.02	0.02	0.02	0.02
8	Silver (Ag)	APHA-3113 (B)	<0.01	<0.01	<0.01	<0.01	<0.01	<0.01	<0.01	<0.01	<0.01
9	Sulphate (SO4)	IS:3,025 (P-24):1986	3.21	1.32	<0.01	6.88	1.85	<0.01	1.46	<0.01	<0.01
10	Fluoride (F)	IS:3,025 (P-60):2008	1.02	2.89	1.10	3.01	3.45	1.90	2.15	1.68	1.61
11	Lead (Pb)	APHA-3110:2017	0.28	0.01	0.03	0.32	0.012	0.06	0.02	0.05	0.05
12	Manganese (Mn)	APHA-3110:2017	0.42	0.68	<0.01	0.31	0.08	<0.01	0.13	<0.01	<0.01
13	Calcium (Ca)	IS:3,025 (P-40):1988	24	24	24	48.0	40	52	24	40	38
14	Magnesium (Mg)	IS:3,025 (P-44):1988	14.67	6.12	17.15	29.34	22.05	31.85	7.35	12.25	13.15
15	Sodium (Na)	IS:3,025 (P-45):1993	1,124	1,035	1,158	2,510	2,395	3,805	977	888	914
16	Potassium (K)	IS:3,025 (P-45):1993	5.2	9.3	9.3	8.9	33.8	18.7	11.6	11.80	11.81
17	Nitrate (NO3)	IS:3,025 (P-34):1988	13.11	7.12	2.65	28.12	9.67	4.26	5.56	4.57	5.68

The physicochemical characterization of the coal, which includes the proximate and ultimate analysis, was previously performed (Chawla et al., 2023). The coal rank was found to be high volatile ‘A’ bituminous (HVAB) as per ASTM standard. High volatile bituminous coal can also be considered to produce biogenic methane which indicates the presence of potential biogenic consortia when stimulated with optimized nutrient formulation (Fallgren et al., 2013).

#### 3.2.2 CBM gas and water production trends

**FIGURE 3:**
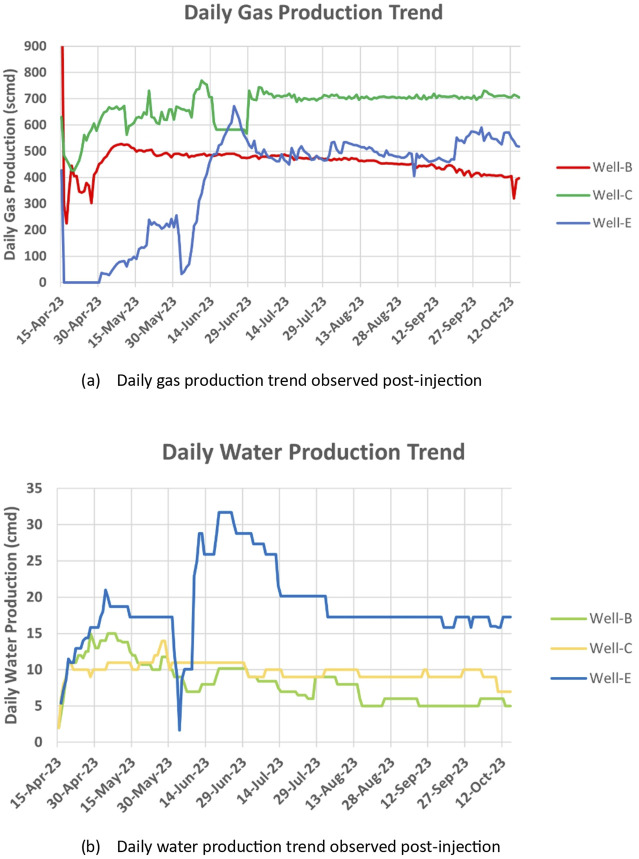
Production trends observed post-stimulation in the study wells.

The gas and water production of the wells was observed for 6 months after the implementation of the bio-stimulation process. The wells were dewatered before observation. Jones et al. (Jones et al., 2013) hypothesized that dewatering the well is important as it increases the desorption of methane gas from coal seams by decreasing the hydrostatic pressure, thereby improving the gas flow rate. The results of this study performed in the Powder River Basin supported the idea that coal bed dewatering may also promote biogenic methanogenesis by partially oxidizing the structural organics in coal after the anerobic conditions are restored. Real-time data acquisition of important well parameters such as CBM gas production, temperature and pressure was performed via a digital platform connected to an online gas flowmeter placed on the well pad. Figure 3 shows the production trends observed in the studied wells post-injection. A significant increase in the gas production from the well was observed. The average gas production of Well-B, Well-C and Well-E during the initial 6 months after the incubation period was 461.38 standard cubic meters per day (scmd), 670.22 scmd, and 385.19 scmd respectively, while the average gas production in 3 months, which was earlier established as the baseline before nutrient injection, was observed as 117.04 scmd, 514.225 scmd, and 210.193 scmd respectively. The average water production in Well-B during the initial 6 months after the incubation period was observed as 8 cubic meters per day (cmd) with a maximum of 15 cmd. The average water production in Well-C and Well-E was observed as 19 cmd with a maximum of 32 cmd, and 11 cmd with a maximum of 14 cmd, respectively. A strong correlation between the production and evolution of coalbed water and the daily variation in gas production has been previously observed by Zhao et al., in 2023 (Zhao et al., 2023). The study also concluded that Total dissolved solids (TDS) in coalbed water rise in tandem with every increase in gas output. A similar pattern has been observed in our study, where the TDS recorded post-injection was higher than the TDS recorded by Chawla et al. (Chawla et al., 2023) in the feasibility studies performed pre-injection.

### 3.3 Feasibility assessment of the technology

Microbially enhanced coalbed methane production via biostimulation of indigenous microbial diversity through nutrient addition is a strategy with promising results in various coal seams. By performing coal-to-methane conversion for cleaner energy production, methane producing microorganisms can reduce the emission of contaminated gases and partially replace the direct use of coal as traditional fossil fuel, thereby providing an advantage of environmental protection (Niu et al., 2024). This optimized technology can be a cost-effective alternative to conventional CBM extraction methods (Bhatt and Tao, 2020), with lower energy requirements, and capability to prevent the formation of by-products toxic to the microbial community, as opposed to the chemical treatment methods (Aworanti et al., 2023). In the current study, the volume of nutrients injected in the CBM wells was 250 m^3^ per well, based upon the coal seam thickness of the reservoir (6.5 m^3^/m of coal seam). The cost of the nutrient media was Rs 20/L (0.23 USD/Litre). The total operational cost of the field application was recovered in a short payback period of 3 months, with substantially enhanced gas production, making it an economically feasible and sustainable process.

### 3.4 Molecular and stable carbon isotope analysis

**FIGURE 4:**
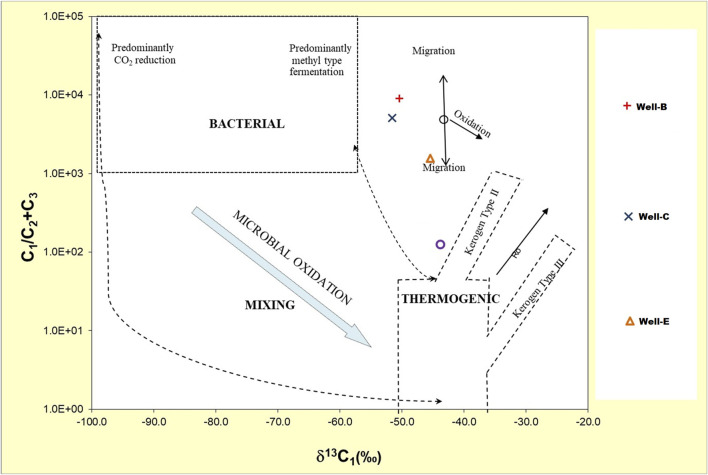
Bernard plot representing the genetic origin of the CBM gas from the Raniganj reservoir after biostimulation, based on the dryness of gas (Bernard et al., 1978).

**FIGURE 5:**
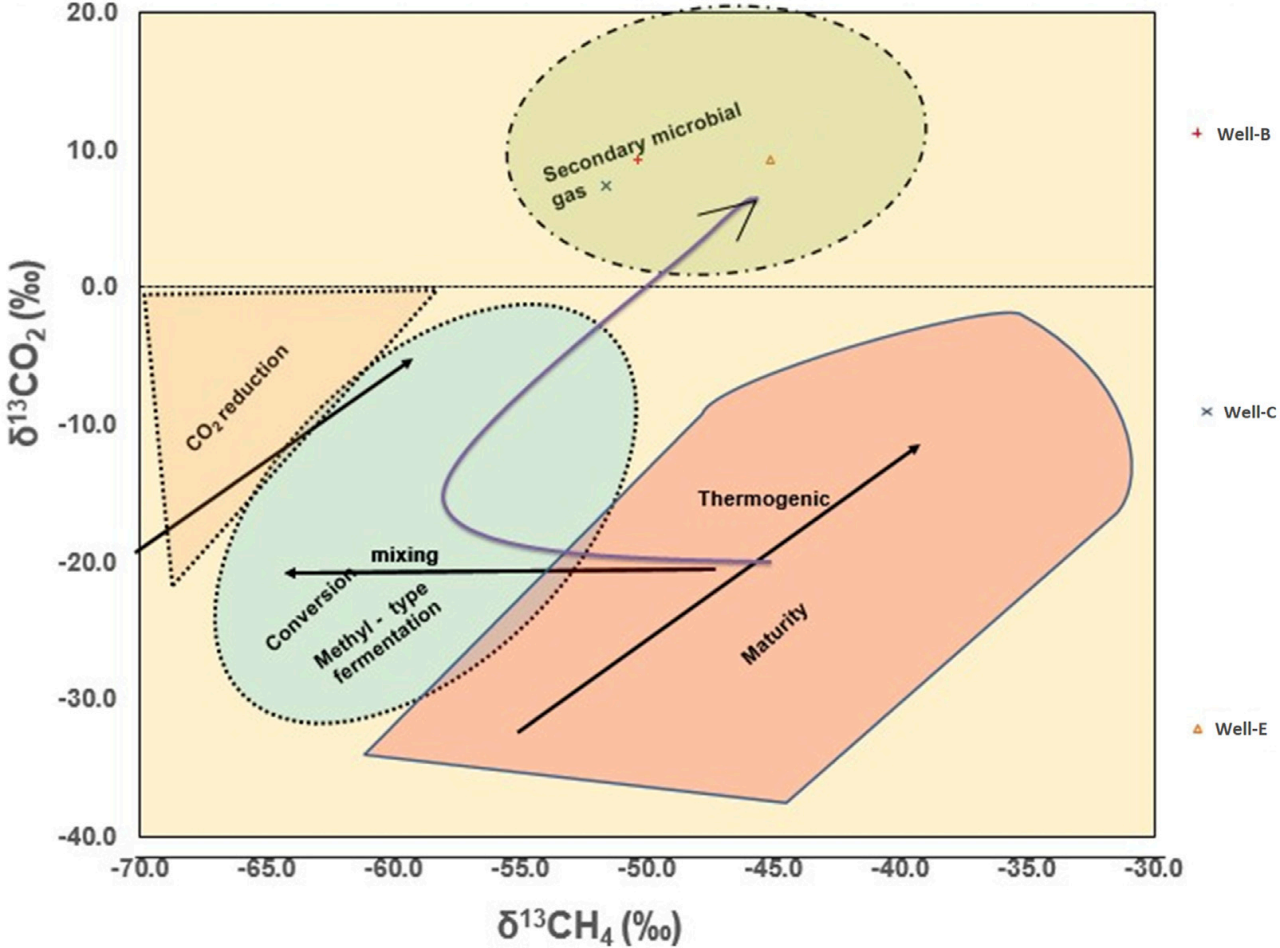
Cross-plot of δ^13^C_1_ and δ^13^CO_2_ showing secondary altercation of gas confirming the occurrence of secondary microbial gas (from [20] and [29]).

The Geochemical analysis of the post microbial stimulation job gas samples collected from the studied wells in the Raniganj field was performed via stable carbon isotope study. The source of the gas can be identified by analyzing the carbon isotopic fingerprints (^δ13^C-CH_4_) generated by thermogenic and biogenic CH_4_ (Prinzhofer and Battani, 2003). The levels of thermogenic CH_4_ carbon stable isotopes range from −50% to −20%, while those of biogenic CH_4_ can have ^δ13^C values between −110% and 40% (Whiticar, 1999). The molecular and isotopic compositions of the gas produced after nutrient treatment indicate mixed origin (Table 4) as the δ^13^C_1_ value of the gas observed in Well-B, Well-C, and Well-E is 50.4%, 51.6%, and 45.1% respectively. The gas is dry in nature in all wells. Methane makes up the majority of microbial gas, with trace amounts of ethane and Propane. The ratio of methane to the sum of ethane and propane (C_1_/C_2_+C_3_), also known as gas dryness index, is one of the most widely used diagnostic geochemical parameters for differentiating gas origin (Golding et al., 2013). The dryness ratio of gas sampled from Well-B, Well-C, and Well-E is 9036.5, 5075, and 1,026.1 respectively. Dryness ratios above 10^3^ are generally regarded to be indicative of microbial methane, whereas values below 10^2^ are indicative of thermogenic methane (Bernard et al., 1978). The Bernard plot based on this ratio (Figure 4) has been widely used as a classification diagram of the genetic origin of natural gas (Bernard et al., 1976; Milkov and Etiope, 2018; Ni et al., 2021; Westerholm et al., 2016). The presence of isotopically heavier carbon dioxide in the studied gases also corroborates mixed origin as microbes preferentially consume ^12^C over ^13^C (Kinnon et al., 2010). A cross plot of δ^13^C_1_ and δ^13^CO_2_ (Figure 5) provides carbon isotope fractionation value(α), where α=(1,000+δ^13^C-CO_2_)/(1,000+ δ^13^C-CH_4_). α values between 1.03 and 1.06 denote acetate and methyl-based fermentation, while α values between 1.06 and 1.09 denote CO2 reduction pathway of methanogenesis (Bishanga and Qiang, 2021). α values of 1.0628, 1.0622, and 1.0568 of the CBM well samples from Well-B, We-C and Well-E respectively, show secondary altercation of gases, indicating the occurrence of secondary microbial gas via CO2 reduction (Milkov, 2011; Whiticar, 1999).

**TABLE 4:** Molecular and stable carbon isotopic compositions of CBM gas collected from the candidate CBM well post biostimulation.

Parameters		Results		
		Well-B	Well-C	Well-E
Chemical Composition (% Mol)	C_1_	98.73	98.31	96.41
	C_2_	-	0.02	0.07
	C_3_	0.01	0.00	0.01
	iC_4_	-	0.00	0.04
	nC_4_	0.01	0.00	0.02
	iC_5_	-	0.00	0.01
	nC_5_	0.01	0.00	0.00
	C_6_	0.01	0.00	0.02
	C_2+_	0.04	0.02	1.25
	CO_2_	0.52	0.56	1.18
	N_2_	0.7	1.1	1.1
Stable Carbon isotopic values (*δ* ^13^C%)	C_1_/(C_2_+C_3_)	9036.5	5075.0	1,026.1
	*δ* ^13^C_1_	−50.4	−51.6	−45.1
	*δ* ^13^C_2_	-		−30.6
	*δ* ^13^C_3_	-		−29.3
	*δ* ^13^CO_2_	9.3	7.4	9.2

### 3.5 Cultivation-independent assessment of the microbial community of post-injection formation water

The approach to the process of CBM enhancement that has been studied the most is biostimulation through nutrient amendment. A thriving indigenous consortium of microbes with the ability to degrade coal and produce methane must exist in order to apply biostimulation techniques *in-situ*. Therefore, the microbial diversity of the candidate CBM wells in the Raniganj coal-seam reservoir was studied prior to the implementation of the biostimulation process (Chawla et al., 2023), and a suitable nutrient media was formulated. Several studies focused on biostimulation have utilized methanogenic substrates such as acetate, CO_2_, and H_2_ (Harris et al., 2008; Jones et al., 2013). While adding these substrates could encourage methanogens to generate methane, the main objective of microbial stimulation is to stimulate coal-dependent methanogenesis. In order to break down coal and provide intermediate products that methanogens may use to convert it to methane, it has been proposed that MeCBM injections should target the colonisers and degraders of coal (also known as “first biters”), rather than just the methanogens (Schlegel et al., 2013). This has been recently achieved in a field study by Barnhart et al., in 2022 (Barnhart et al., 2022). Yeast extract was injected into the reservoir for *in-situ* biostimulation, and the isotopic study results indicated that it potentially stimulated the primary and secondary coal-degrading microbes that provided co-metabolites to methanogens.

**FIGURE 6:**
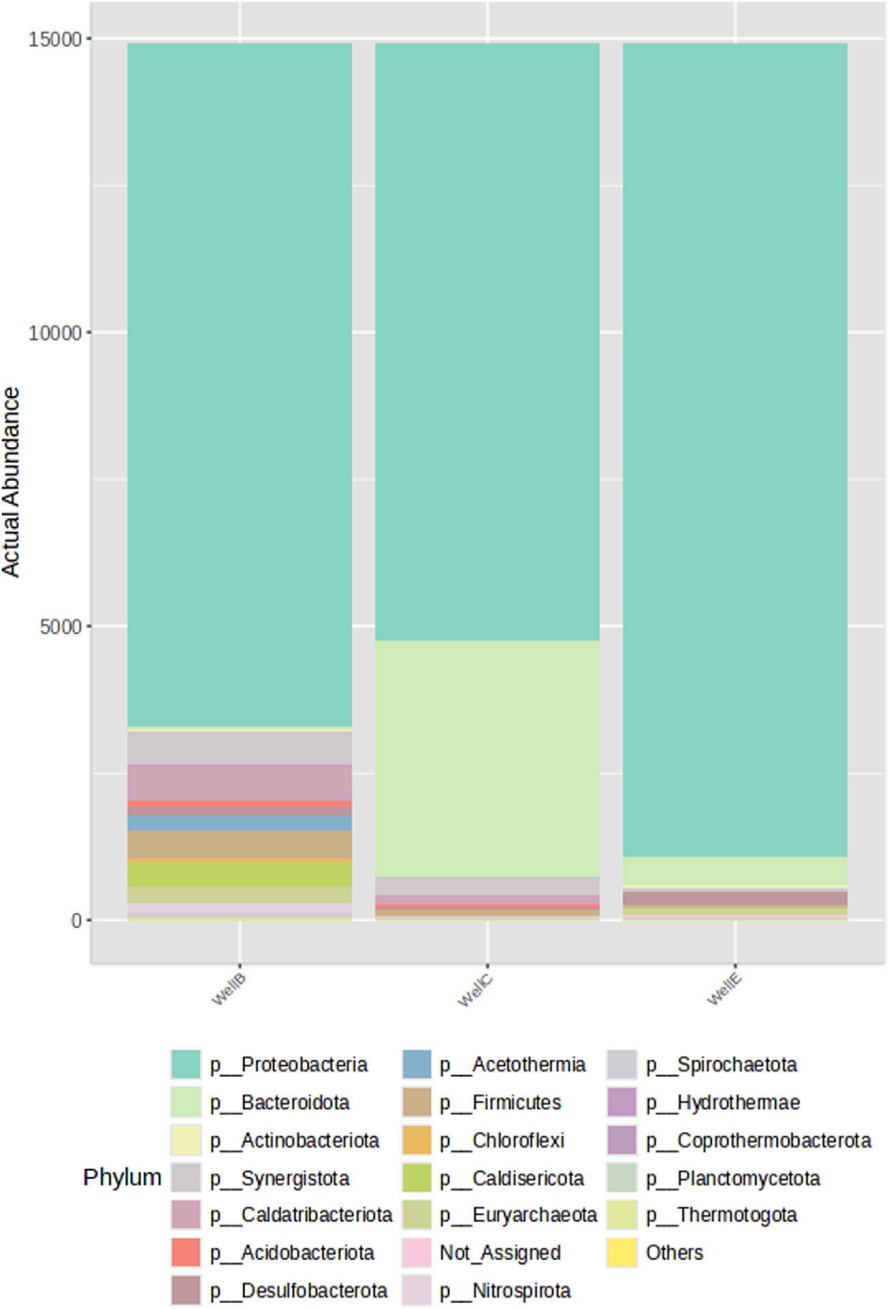
Stacked bar plot representing the percentage abundance assessed by 16S rRNA-gene amplicon sequencing at phylum-level.

Proteobacteria makes up the majority of the bacteria found in the formation water sample taken from the bio-stimulated CBM Well-B, Well-C and Well-E followed by other dominating phyla such as Caldatribateriota, Synergistota, Firmicutes, Caldisericota, and Aciderothermia. The presence of phyla including Bacteroidota, Chloroflexi, Nitrospirota, Acidobacteriota, Desulfobacterota, and Actinobacteriota is also seen in all CBM wells. (Figure 6). Niya et al. (2024) assessed the microbiome composition in anaerobic digestion systems and found that Caldatribacteriota, Synergistota, Firmicutes, Bacteroidota, and Chloroflexi were the dominant phyla found in a continuously stirred tank reactor (CSTR) in which the methane output, metabolic transformation and microbial response were studied in relation to the performance of anaerobic co-digestion of organic compounds. Caldatribacteriota, found in geothermal systems, petroleum reservoirs, wastewater treatment facilities and anaerobic digestors, produces hydrogen and acetate via fermentation (Dodsworth et al., 2013). Firmicutes are believed to be responsible for the demethylation of aromatic compounds, a crucial step in the cleavage of aromatic compounds (Strąpoć et al., 2008), and were also seen in the formation water from the Jharia basin in an investigation conducted by Singh et al. (2012). Similar to the present investigation, Spirochaetes of the phylum Spirochaetota predominate the Jharia coal reservoir, and the coal bed of the Illinois basin (Strąpoć et al., 2008). In the Powder River Basin, Spirochaetes were a part of the methanogenic consortia, as demonstrated by the study done by Green et al. (2008). Firmicutes also dominated the bacterial population in the coal bed methane wells in the Powder River Basin. The archaeal community of the formation water consists of Euryarchaeota, composed of physiologically diverse groups, including methanogens, the most well-researched group. The primary metabolic activities of this group include oxidation of coal hydrocarbons and methanogenesis (Beckmann et al., 2019).

**FIGURE 7:**
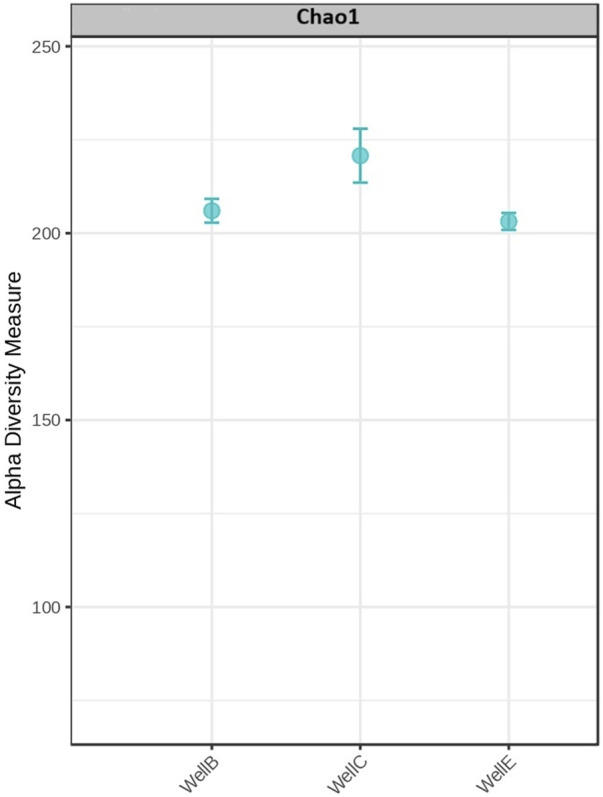
Alpha diversity evaluation at genus level: Chao1 diversity index.

The high abundance of microbial phyla observed suggests a strong presence of microorganisms involved in early anaerobic digestion stage, particularly hydrolysis and acidogenesis. The diversity richness in the samples has been analyzed with the Alpha diversity analysis using the Chao 1 diversity index (Figure 7).

**FIGURE 8:**
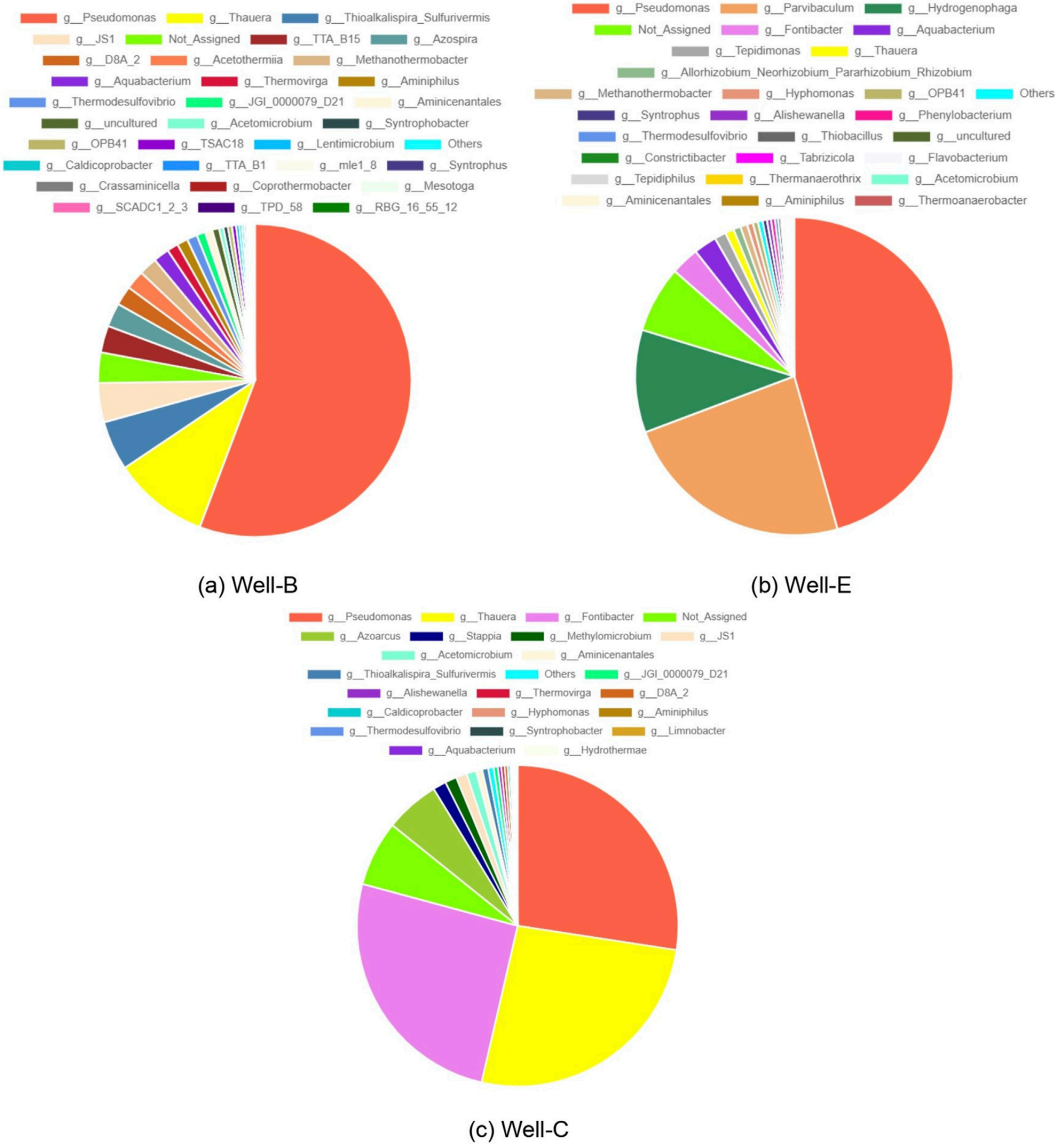
Pie chart depicting microbial diversity at the genus level in the formation water analyzed post-injection in **(a)** Well-B, **(b)** Well-E, and **(c)** Well-C.

Figure 8 represents the microbial diversity at genus level in the formation water samples collected post-injection. The microbial diversity of pre-injection formation water was analyzed by Chawla et al. (Chawla et al., 2023) as part of the feasibility study and strategization for field-scale biostimulation for enhanced biogenic methane production. The prevalence of *Pseudomonas* in post-injection formation water samples of Well-B, Well-C and Well-E as compared to its proportion in the pre-job formation water samples is of utmost importance as it plays a vital role in coal degradation, which is the foremost target of the biostimulation process. *Pseudomonas* has the capability to break down alkanes, alkenes, and polycyclic aromatic hydrocarbons (PAH) into formate, lactate, acetate, ethanol, and CO2. Additionally, it can aid in the breakdown of PAH by producing biosurfactants, which improve solubility by reducing surface and interfacial tensions between coal molecules (Colosimo et al., 2016; Davis and Gerlach, 2018; Singh and Tripathi, 2013). *Pseudomonas* is also well known to play a crucial role in the depolymerization of lignin (Rouches et al., 2021). The prevalence of *Pseudomonas* has also been noted in the Erlian Basin, China by Fu et al., in 2023 (Fu et al., 2023). *Thauera Syntrophobacter*, S*ulfurivermis*, *are* also found to be in dominance in the post-injection samples of Well-B and Well-C, while their abundance in the pre-job sample was found to be significantly low. Similarly, Azospira, which was found to be low in abundance in pre-injection samples, is abundant in the post-injection samples of Well-B. Particularly, *Pseudomonas*, *Thauera*, and *Azospira are* regarded as key PAH degraders under nitrate reducing conditions, while *Syntrophobacter* is a syntrophic propionate-degrading bacteria, and S*ulfurivermis* degrades PAH under sulfate-oxidizing conditions (Torres-Herrera et al., 2024). A similar study performed by Beckmann et al. (Beckmann et al., 2019) in a gas-free coal seam reservoir in New South Wales, Australia, also involved the bio-stimulation of coal bed methane via nutrient addition. It showed results resembling that of the present study, as the abundance of hydrocarbon degrading bacteria-affiliated with *Pseudomonas*, *Thauera*, *Azospira*, and *Syntrophobacter*-increased after the nutrient amendment. These lineages exhibit a global distribution, as evidenced by their documented presence in coalbed seams across the Powder River Basin, Ruhr Bain, Alberta Basin, Illinois Basin, Ishikari Basin, Waikato coalfields, and deep-sea coalbeds (Barnhart et al., 2013; Beckmann et al., 2011; Gründger et al., 2015; Inagaki et al., 2015; Penner et al., 2010; Shimizu et al., 2007). The most prevalent bacterial phyla in the western coalfields of Australia were Firmicutes, Proteobacteria, Bacteroidetes, and Spirochaetes.

Genus *JS1* of the candidate phylum Atribacteria, and genus *D8A*_*2* belonging to phylum Firmicutes were also found to be abundant in the post-injection samples of Well-B and Well-C. D8A_2 is a syntrophic acetate-oxidizing bacteria (C. Li et al., 2022). *JS1* is among the few bacterial lineages that are typically found in deep surface environments including oil reservoir formation water as well as stable hydrocarbon-degrading enrichment cultures that are derived from oil reservoirs (Y.-F. Liu et al., 2019). The proliferation of *JS1* after *in-situ* biostimulation is highly significant as it has fermentative potential using a variety of substrates and is involved in syntrophic acetate oxidation in conjunction with H_2_ and CO_2_ utilizing methanogens (Lee et al., 2018). Genus *TTA*_*B15*, a member of the class Caldisericia, which was also previously found to be in insignificant proportion, is present in the post-injection sample of Well-B, and is a known hydrocarbon-degrading bacteria typically found in petroleum reservoirs (Tatar, 2018). It has also been found in Daqing oil field, China (F. Zhang et al., 2010). *Thermovirga,* of class Synergistia, a fermentative hydrocarbon-degrading bacterium (Mbadinga et al., 2011) has been found to be more abundant in the post-injection samples of Well-B and Well-C. Similarly, an increase in the abundance of *Aminiphilus*, an amino acid degrading bacterium (Díaz et al., 2007), and *Aquabacterium,* known to be involved in biodegradation of oil (Q. Wang et al., 2019) has also been observed in the post-injection samples of all three wells. Increased abundance of an unclassified member of *Acetothermia* has been observed in Well-B. The presence of *Acetothermia* has been previously detected in anaerobic digestors (Nobu et al., 2015), suggesting that it may be specifically adapted to this environmental niche, and contributes to the process of converting organic matter into biogas (Hao et al., 2018).

The abundance of archaeal genus *Methanothermobacter* in the post-injection formation water samples of all three CBM wells as opposed to the pre-injection formation water samples is especially noteworthy. It is a well-known thermophilic, CO_2_ reducing, hydrogenotrophic methanogen, i.e., it exclusively utilizes H_2_+CO_2_ as a substrate in the process of methanogenesis (Smith et al., 1997). It was also found as the dominant archaeal group in the Jharia coal basin (Singh et al., 2012), and the Bokaro coal field (Sahu et al., 2024). Investigations in the Powder River Basin, USA, and the coal reservoir of the Ruhr Basin, Germany, which is a biogenic methane-producing source, also revealed the presence of *Methanothermobacter* (Flores et al., 2008). Abundance of Methanothermobacter in the pre-injection and post-injection samples and Methanobacterium in the pre-job samples (Chawla et al., 2023) suggests that the hydrogeotrophic methanogenesis pathway dominates in the Raniganj reservoir.

**FIGURE 9:**
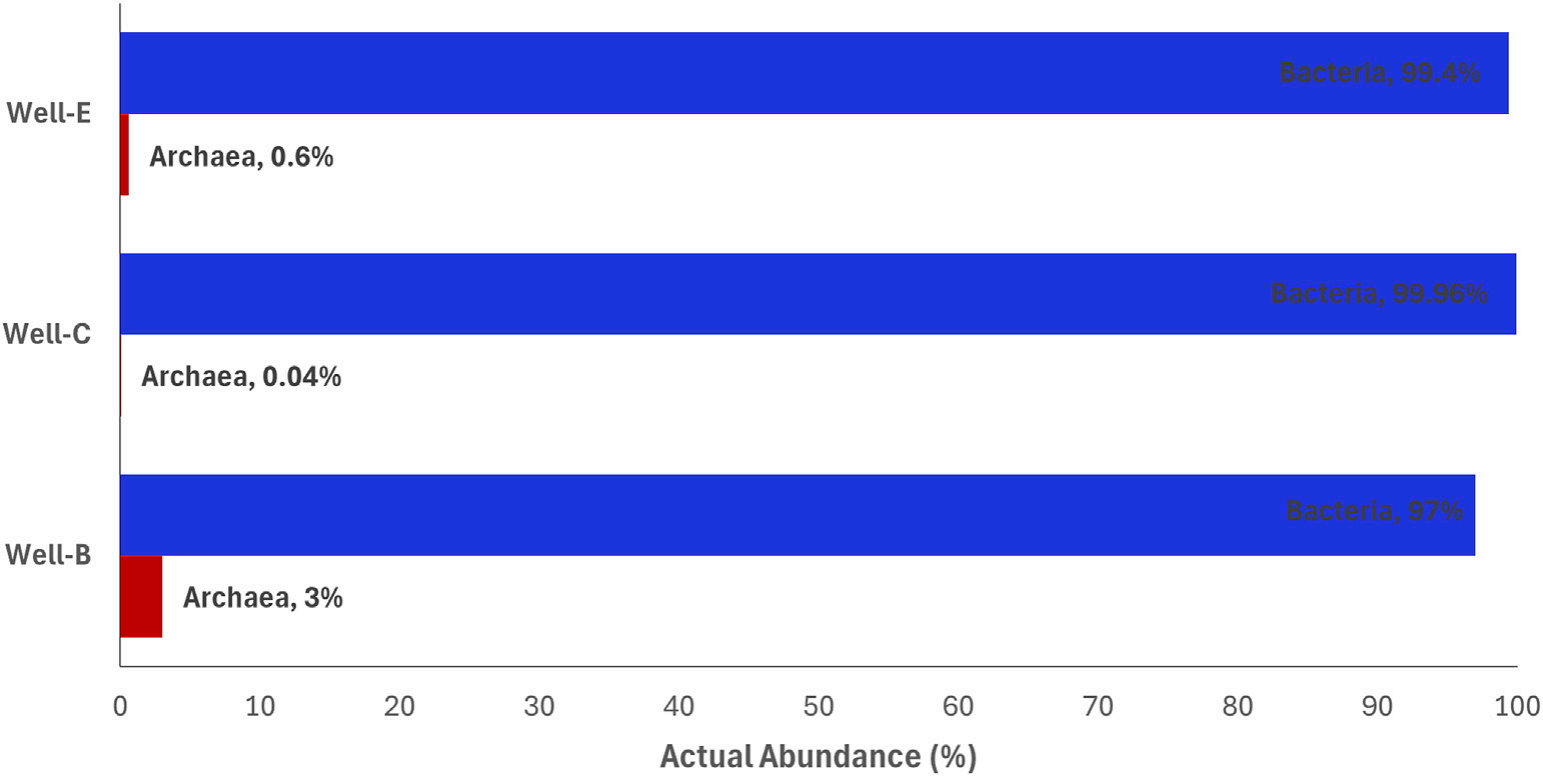
Relative abundance of bacteria vs. archaea in samples post-injection, analyzed on the platform Metagenassist.

Despite the crucial role of methanogenic archaea in methanogenesis being widely known, the limited archaeal abundance in the post-injection samples (Figure 9) is not surprising as the nutrient stimulation was designed to target hydrolytic and fermentative bacteria responsible for breakdown of coal into simple organic substrates, a rate-limiting step in the process of methanogenesis. The relative abundance of bacteria and archaea assessed in various methane producing coal reservoirs has shown that the archaeal abundance was significantly less than the bacterial abundance (Davis et al., 2018; Green et al., 2008; Gründger et al., 2015; B. Liu et al., 2022; J; Zhang et al., 2015).

While the metagenomic analysis provides a comprehensive understanding of the microbial ecology of the selected Raniganj CBM wells by predominantly representing the planktonic microbial communities present in the formation water of the wells, it does not take into account the biofilm-producing microorganisms on the coal surface, which may exhibit functional profiles that are key in process of degradation of organic compounds. Beckmann et al. (2019) performed the characterization of the microbial communities in the coal surface of a coal seam and the effect of *in-situ* biostimulation, and found that methanogenic archaea made up the major proportion of the biofilm producing communities on coal surfaces. Post nutrient amendment results showed a high abundance of *Geobacter uraniireducens*, and sulfate reducing bacteria within the orders *Desulfovibrionales* and *Desulfobacterales*.

### 3.6 Predictive functional analysis of the post-injection microbial abundance

**FIGURE 10:**
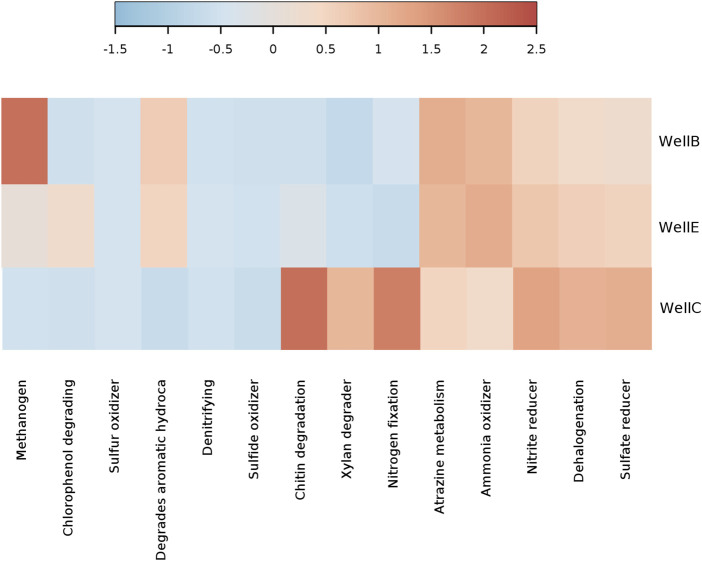
Relative function abundance predictions of microbial communities in post-injection CBM wells.

Functional community profiles of the taxa identified by 16s rRNA sequencing were predicted to offer insights on the microbial metabolism of the microbial diversity enriched in the post-injection formation water samples of the studied CBM wells in the Raniganj coal reservoir. Understanding the metabolic indicators involved in the microbial processes that particularly contribute to coal biodegradation and biogenic methane generation was crucial to optimize the MeCBM process. The heatmap of relative function abundance prediction (Figure 10) shows high methanogenic activity, particularly in Well-B and Well-E, confirmed by higher abundance of Methanothermobacter in these wells. A higher abundance of aromatic hydrocarbon degrading metabolisms was predicted in Well-B and Well-E. The presence of atrazine metabolizing functions is abundant in all wells. Nitrogen fixation, xylan degrading metabolisms and chitin degrading metabolisms predicted a higher relative abundance in Well-C as compared to Well-B and Well-E, similar to nitrate reducing and sulfur reducing metabolisms.

## 4 Conclusion

A field trial of *in-situ* biostimulation via nutrient injection was reported for the first time in CBM wells of the Raniganj coal-seam reservoir in the Gondwana Basin, India. The formation water analyzed bi-monthly post-injection showed a stable physicochemical profile of the wells throughout the observation period. Upto four-fold increase in the production of CBM gas from the treated wells was observed, and the production levels were maintained 6 months after the incubation period. Establishing a control baseline gas production value based on the production data of 3 months prior to the field trial allowed us to definitively attribute the increase in gas production in the CBM wells to the effects of the nutrient treatment. A favorable impact on the indigenous microbiome of the formation water was observed upon the assessment of changes in the microbial communities and their abundance after *in-situ* biostimulation in comparison to the microbial diversity found in the formation water samples collected before the implementation of the biostimulation process. The targeted communities, particularly hydrocarbon-degrading bacteria, proliferated after nutrient injection. A surge in the abundance of key coal hydrocarbon degraders including *Pseudomonas*, *Azospira*, *Thauera*, and *Sulfurivermis*, amongst other abundant genera in the formation water samples was observed. The notable presence of methanogenic archaea *Methanothermobacter* in all post-injection formation water samples as opposed to its significantly low abundance in the pre-injection formation water samples suggests that hydrogenotrophic methanogenesis is the primary pathway of biogenic methane production that occurred as a result of *in-situ* biostimulation. The successful implementation of the MeCBM technology and the observed upsurge in the gas yield demonstrates its potential on a large scale, which may help the oil and gas industry contribute towards the nation’s vision of being a gas-based economy and achieve the sustainable development goal (SDG-7) of affordable and clean energy.

## Data Availability

The datasets presented in this study can be found in online repositories. The names of the repository/repositories and accession number(s) can be found in the article/Supplementary Material.
