# Supplementary material for: In-situ bio-stimulation for enhanced biological methane production and its effect on the microbiome of CBM wells in Raniganj block, India
Source: Front Bioeng Biotechnol. 2025 Jun 27;13:1571653. doi: 10.3389/fbioe.2025.1571653 (PMC12285592; doi:10.3389/fbioe.2025.1571653)
Supplement: Supplementary file 1 [file DataSheet1.docx]

***Supplementary Material***

**1. The effect of coal on the stimulation of methane-producing microbial consortium**

To ascertain whether the indigenous microbial population of the studied CBM well would utilize coal as a carbon source for biological methane production was tested on a lab-scale in the initial phase of the study during media modification experiments in the feasibility studies. Experimental sets included modified nutrient media with coal and modified nutrient media without coal. At this stage, the modified nutrient media included both yeast extract and sodium bicarbonate along with other media components, as previously tested by Chawla et al. [1]. The pH was then adjusted to 7.0 ± 0.2. 100 μL of Resazurin was added to the media as an oxygen indicator. The media was boiled for 10 min and allowed to cool down while being purged with nitrogen to remove the dissolved oxygen. The remaining dissolved oxygen was then subsequently removed by adding 0.5 g L-cysteine hydrochloride, making the environment of the media completely oxygen-free. A volume of 30 mL media was dispensed into 60 mL anaerobic serum bottles. 1% (w/v) coal was added, and the bottles were sparged again with nitrogen to maintain an anaerobic environment. The bottles were sealed with butyl rubber stoppers and crimped with aluminum caps. The sealed and pressurized bottles were autoclaved before inoculation for 15 min at 121°C. Followed by inoculation with 10% (v/v) formation water collected from the study wells, the bottles were incubated at 55°C for 30 days. Methane generation in the incubated bottles was monitored at day 30 by collecting 0.5 mL of headspace gas samples from the serum bottles via a gas-tight syringe and quantifying the headspace gases, via gas chromatography using the Agilent 7890A GC system TCD detector. All experiments were run in a triplicate set. The data points are the average of triplicate±standard deviation (<5% of average).

Low methane levels were observed in nutrient-only sets. While yeast extract and sodium bicarbonate may have contributed to biological methanation in both sets, the sets with coal showed higher long-term production of methane as compared to sets without coal (Supplementary Fig. 1), indicating the utilization of coal also by the microorganisms for methane generation. Similar results have been found in previous studies [2], [3], where nutrient-only sets showed low yields of methane, and it was thus concluded that it is unlikely that the methanogens primarily utilized yeast extract as carbon source and then switched to the other predominant carbon source, coal, after the injected yeast extract was depleted. It is more likely that the production of certain metabolites during the utilization of yeast extract allowed the microbes to break down coal and generate methane gas via anaerobic digestion [4].


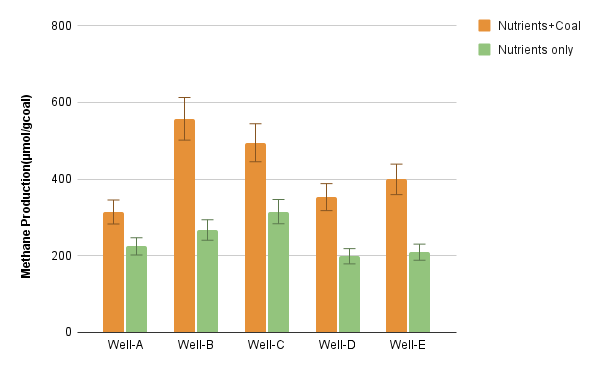


**Supplementary Fig. 1:** Methane production rates in nutrients-only and nutrients+coal sets

**2. Single factor analysis of chemical factors influencing methane production**

A single factor experiment was performed during the media optimization studies to identify the essential media components for microbial methane production. The effect of each component in the modified MPB media composition was analyzed during feasibility studies conducted by Chawla et al. [1]. The current data represents the effect of yeast extract and sodium bicarbonate on microbial methane production. Set-1 represents nutrient media minus sodium bicarbonate, set-2 represents nutrient media minus yeast extract, and set-3 represents nutrient media including all components. The media sets were prepared in anaerobic serum bottles. 1% (w/v) coal was added, and the bottles were sparged with nitrogen to maintain an anaerobic environment, followed by inoculation with 1% (v/v) formation water collected from the study wells. The bottles were incubated at 55°C for 30 days. Methane generation in the incubated bottles was monitored at day 30 by collecting 0.5 mL of headspace gas samples from the serum bottles via a gas-tight syringe and quantifying the headspace gases, via gas chromatography using the Agilent 7890A GC system TCD detector. All experiments were run in a triplicate set. The data points are the average of triplicate±standard deviation (<5% of average).

It was observed that the methane production rate was the lowest in the absence of yeast extract in the nutrient composition. The production was also significantly low in the absence of sodium bicarbonate, as compared to the production in the set with both yeast extract and sodium bicarbonate present in the nutrient composition (Supplementary Fig. 2). It was concluded that the contribution of yeast extract and sodium bicarbonate was significant for high production rates of methane, and the media composition used in set-3 was optimal for achieving the goal of the current study that was to commercially enhance the methane production in CBM wells of Raniganj.


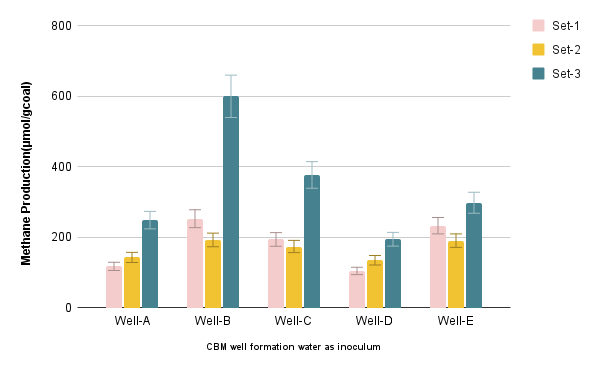


**Supplementary Fig. 2:** The effect of chemical factors on methane production rate. Set-1 represents nutrient media minus sodium bicarbonate, set-2 represents nutrient media minus yeast extract, and set-3 represents nutrient media including all components.

**2.1. Discussion**

As the aim of the study was to enhance methane production at a commercial scale via biostimulation of indigenous microbial community present in the CBM wells of the Raniganj field, the candidate wells were studied for the presence and relative abundance of hydrocarbon-degrading and methane-producing microbes prior to biostimulation. However, the gas production rates of these wells were not at par at the time. Therefore, it was necessary that the coal degrading microbes adsorbed on the coal present in the reservoir were first stimulated, for which the amendment of nutrients such as yeast extract and sodium bicarbonate, among others, was crucial. Coal matrices are made up of complex aliphatic and aromatic hydrocarbons and lack nutrients required by microorganisms, such as proteins and inorganic salts. As a result, protein and other resources required for microbial metabolism must be provided to the microorganisms for them to produce methane via anaerobic digestion of coal [4]. Yeast extract is one of the most widely utilized nitrogen sources. The principal constituents of yeast extract are crude protein, total nucleotide, amino nitrogen, $\beta$-glucan, and mannan oligosaccharide. Microorganisms can grow more efficiently if their rate of nutrient digestion and absorption is accelerated by the yeast nucleotides present in yeast extract and can subsequently utilize coal for methane production [5]. The solubility and biodegradation of coal are enhanced by the ability of yeast extract to chelate certain metal cations, as demonstrated by earlier studies [6], [7].

Furthermore, observations in the supplementary fig. 1 may also be noteworthy as the nutrients-only sets yielded lower levels of methane than the nutrient media+coal sets, indicating the utilization of coal also by the microorganisms for maximum methane generation. In both cases, yeast extract and bicarbonate were present in the nutrient media. This indicates that organic compounds, not only in the added nutrients, but also in the coal were converted to methane.

**3. Rarefaction curve**

A rarefaction curve was plotted between the species richness and the sequence sample size.

**
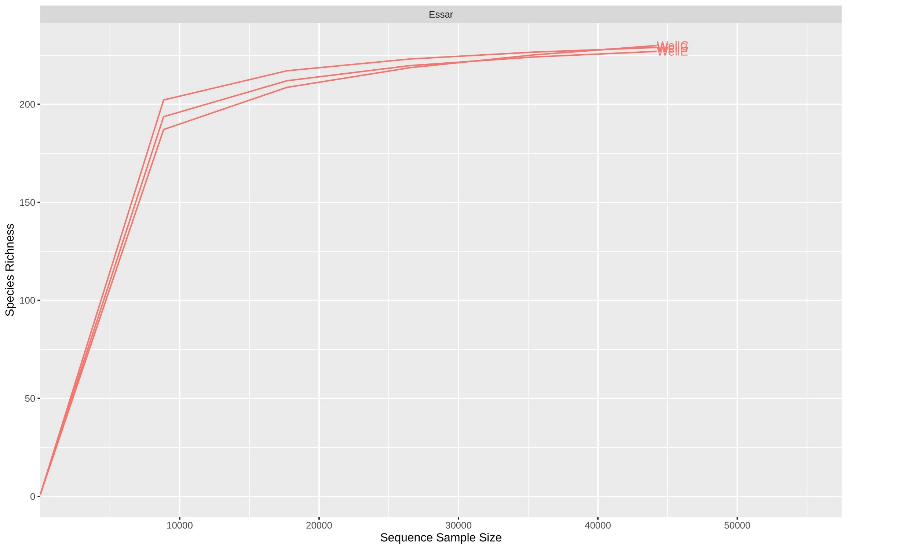
**

**Supplementary Fig. 3:** Rarefaction curve depicting sequence richness and depth of the samples

**References**

[1] M. Chawla *et al.*, “Culture-independent assessment of the indigenous microbial diversity of Raniganj coal bed methane block, Durgapur,” *Front. Microbiol.*, vol. 14, 2023, doi: 10.3389/fmicb.2023.1233605.

[2] K. J. Davis, S. Lu, E. P. Barnhart, A. E. Parker, M. W. Fields, and R. Gerlach, “Type and amount of organic amendments affect enhanced biogenic methane production from coal and microbial community structure,” *Fuel*, 2018, doi: https://doi.org/10.1016/j.fuel.2017.09.074.

[3] E. P. Barnhart *et al.*, “In Situ Enhancement and Isotopic Labeling of Biogenic Coalbed Methane,” *Environ. Sci. Technol.*, vol. 56, no. 5, pp. 3225–3233, 2022, doi: 10.1021/acs.est.1c05979.

[4] S. Y. Park and Y. Liang, “Biogenic methane production from coal: A review on recent research and development on microbially enhanced coalbed methane (MECBM),” *Fuel*, vol. 166, pp. 258–267, 2016, doi: 10.1016/j.fuel.2015.10.121.

[5] J. Zhang, Y. Liang, R. Pandey, and S. Harpalani, “Characterizing microbial communities dedicated for conversion of coal to methane in situ and ex situ,” *Int. J. Coal Geol.*, vol. 146, pp. 145–154, 2015, doi: 10.1016/j.coal.2015.05.001.

[6] E. J. P. Jones, M. A. Voytek, M. D. Corum, and W. H. Orem, “Stimulation of Methane Generation from Nonproductive Coal by Addition of Nutrients or a Microbial Consortium,” *Appl. Environ. Microbiol.*, vol. 76, no. 21, pp. 7013–7022, 2010, doi: 10.1128/AEM.00728-10.

[7] S. H. Harris, R. L. Smith, and C. E. Barker, “Microbial and chemical factors influencing methane production in laboratory incubations of low-rank subsurface coals,” *Int. J. Coal Geol.*, vol. 76, no. 1–2, pp. 46–51, 2008, doi: 10.1016/j.coal.2008.05.019.
